# TLR3-Induced Placental miR-210 Down-Regulates the STAT6/Interleukin-4 Pathway

**DOI:** 10.1371/journal.pone.0067760

**Published:** 2013-07-02

**Authors:** Shelley E. Kopriva, Valorie L. Chiasson, Brett M. Mitchell, Piyali Chatterjee

**Affiliations:** Department of Internal Medicine, Division of Nephrology and Hypertension, Scott & White Healthcare/Texas A&M Health Science Center, Temple, Texas, United States of America; St. Jude Children’s Research Hospital, United States of America

## Abstract

Several clinical studies have reported increased placental miR-210 expression in women with PE compared to normotensive women, but whether miR-210 plays a role in the etiology of PE is unknown. We reported that activation of TLR3 produces the PE-like symptoms of hypertension, endothelial dysfunction, and proteinuria in mice only when pregnant, but whether TLR3 activation in pregnant mice and human cytotrophoblasts (CTBs) increases miR-210 and modulates its targets related to inflammation are unknown. Placental miR-210 levels were increased significantly in pregnant mice treated with the TLR3 agonist poly I:C (P-PIC). Both HIF-1α and NF-κBp50, known to bind the miR-210 promoter and induce its expression, were also increased significantly in placentas of P-PIC mice. Target identification algorithms and gene ontology predicted STAT6 as an inflammation-related target of miR-210 and STAT6 was decreased significantly in placentas of P-PIC mice. IL-4, which is regulated by STAT6 and increases during normotensive pregnancy, failed to increase in serum of P-PIC mice. P-PIC TLR3 KO mice did not develop hypertension and placental HIF-1α, NF-κBp50, miR-210, STAT6, and IL-4 levels were unchanged. To determine the placental etiology, treatment of human CTBs with poly I:C significantly increased HIF-1α, NF-κBp50, and miR-210 levels and decreased STAT6 and IL-4 levels. Overexpression of miR-210 in CTBs decreased STAT6 and IL-4 while inhibition of miR-210 increased STAT6 and IL-4. These findings demonstrate that TLR3 activation induces placental miR-210 via HIF-1α and NF-κBp50 leading to decreased STAT6 and IL-4 levels and this may contribute to the development of PE.

## Introduction

Preeclampsia (PE) is a vascular disorder that affects 5–8% of all pregnancies and is diagnosed by the onset of hypertension and proteinuria at or after the 20^th^ week of gestation [1]. PE is one of the leading causes of preterm birth as 10% of babies are born before 34 weeks of gestation. Although the pathophysiology of PE is still unknown, a major contributing factor is excessive activation of the maternal immune system and inflammation [2], [3]. One possible cause of the inflammatory state may be excessive activation of Toll-like receptors (TLRs) which are present throughout utero-placental tissues to guard against foreign pathogens [4]–[7]. TLRs recognize exogenous pathogens and endogenous ligands from necrotic tissues and initiate innate immune responses by inducing pro-inflammatory cytokines and type I interferons (IFNα/β) [8], [9]. Out of the many TLRs, double-stranded RNA viruses bind to TLR3 leading to activation of the adaptor molecule TRIF, which is involved in IRF3 and NF-κB activation, and subsequently leads to IFN-β production and the expression of IFN-inducible genes [10]. Several studies link viral infections with a 2-fold increased risk of developing PE [11], [12]. We have reported previously that TLR3 activation is increased in placentas of women with PE and that activating the maternal immune system with the TLR3 agonist poly I:C in pregnant mice leads to PE-like symptoms [13], [14]. TLR3-induced PE mice exhibit increased serum levels of pro-inflammatory cytokines including IFNγ, TNFα, and IL-12, while levels of the anti-inflammatory cytokine IL-4 fail to increase [13]. Clinical studies also reported increased levels of pro-inflammatory cytokines in women with PE and decreased levels of IL-4 [15], [16]. Thus, we sought to determine the molecular mechanism by which IL-4 levels fail to increase during PE.

Several recent studies indicate that miRs may modulate TLR-mediated immune responses including production and release of cytokines and chemokines [17]–[19], expression of adhesion and co-stimulatory molecules [17], [20], and feedback regulation of immune responses [17], [18], [21]. miRs are endogenous, small (19–23 nucleotides long) single-stranded noncoding RNA that suppress gene expression either via translational inhibition or mRNA degradation (or both) and have emerged as key post-transcriptional regulators of gene expression [22]–[26]. Recent studies validated that miRs are abundant in the placenta [27], [28] suggesting a role for miRs in regulating placental gene expression. Dysregulation of miRs are associated with the pathogenesis of several diseases including PE. To date, several microarray-based placental miR profiles have been reported in PE patients [29], [30]. Among them, miR-210 was found in several studies to be elevated significantly in PE women [29], [31]. Whether or not miR-210 expression also increases in our TLR3-induced PE mouse model is unknown. Moreover, expression of miR-210 has been shown to be induced during hypoxia by hypoxia inducible factor-1α (HIF-1α) [32]–[35] and the nuclear factor-κB (NF-κB) p50 subunit [36]. Whether the same transcription factors also regulate miR-210 expression during normoxia remains to be elucidated. Although placental miR-210 expression is increased in PE patients, very few miR-210 targets and their pathophysiological role in PE have been identified so far. The targets which have been identified to date include iron sulfur cluster scaffold homologue (ISCU), Ephrin A3, Homeobox A9 (HOXA9), and more recently hydroxysteroid (17-β) dehydrogenase [36]–[39]. Thus there is a need to identify additional miR-210 targets and disseminate their role in the pathophysiology of PE.

In the present study we hypothesized that TLR3 activation via poly I:C increases placental miR-210 via activation of HIF-1α and NF-κBp50 which suppresses the STAT6/IL-4 anti-inflammatory pathway leading to PE. Here we demonstrate that TLR3 activation induced the expression of miR-210, HIF-1α, and NF-κBp50 in placentas of wild-type mice as well as human CTBs. We further demonstrate that STAT6 is a novel target of miR-210 using overexpression and inhibition studies in human CTBs and that STAT6 in turn modulates IL-4. Additionally, poly I:C-treated pregnant TLR3 KO mice do not exhibit changes in HIF-1α, NF-κBp50, miR-210, STAT6, and IL-4 levels and do not develop PE.

## Results

### TLR3 Induced miR-210 Up-regulation in Pregnant Mice

**Figure 1:**
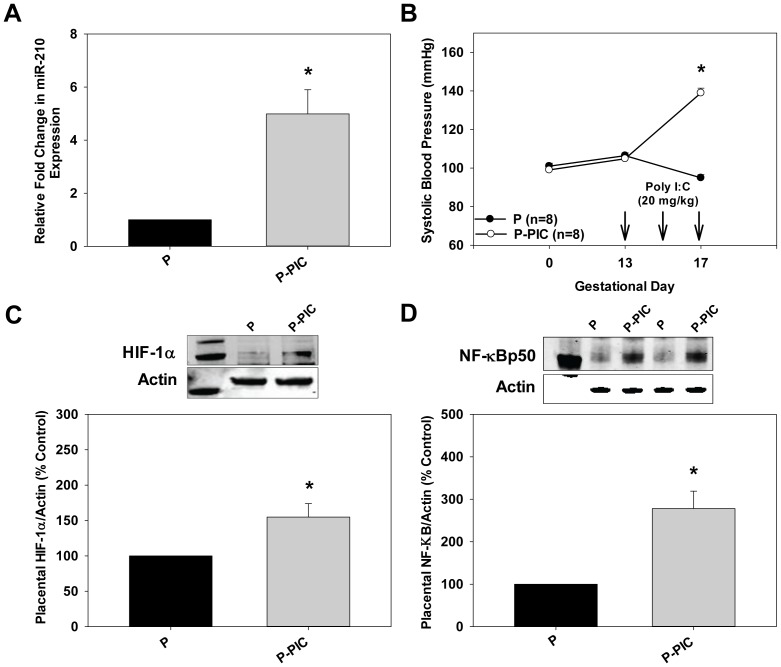
HIF-1α and NF-κB induced miR-210 expression in hypertensive P-PIC mice. A. Total RNA (including small RNAs) was isolated from placentas of both P and P-PIC mice at gestational day 18. miR-210 expression was subsequently determined by qRT-PCR. qRT–PCR of snoRNA 142 was used for normalization. Results are expressed as mean+SEM for 3 independent experiments. *P<0.05 by Student’s t-test. B. Systolic blood pressures in P and P-PIC mice were measured at gestational days 0, 13, and 17 via tail-cuff plethysmography. The results are expressed as mean+SEM with 8 mice in each group. *P<0.05 vs. P. C and D. Immunoblot analyses using anti-HIF-1α and anti-NF-κB antibodies on cell lysates prepared from placentas of P and P-PIC mice at gestational day 18. β-actin was used as a loading control. The first lane in both immunoblots indicate the molecular weight marker. Signals from 3 independent experiments were quantified and expressed as a percentage of P. The results are expressed as mean+SEM for percentage of P for 3 independent experiments. *P<0.05 vs. P.

miR-210 expression is increased in placentas of women with PE. To determine if miR-210 expression is also induced in placentas from P-PIC mice compared to P mice at gestational day 18 we performed qRT-PCR reactions. Compared to control P mice, miR-210 expression increased significantly in P-PIC placentas (4.9 fold, p<0.05, Fig. 1A) and this was associated with increased systolic blood pressure (P: 95±2 mm Hg vs. P-PIC: 139±2 mm Hg, *P*<0.05; Fig. 1B). Previous studies have shown that miR-210 is induced during hypoxia and HIF-1α was identified as a transcription factor that binds to the promoter of miR-210 [32]–[34]. HIF-1α levels were also increased significantly in P-PIC placentas (1.5 fold, p<0.05 vs. P placentas, Fig. 1C) suggesting that HIF-1α likely binds to the promoter of miR-210 under normoxia. Moreover, the NF-kBp50 subunit also binds to the promoter of miR-210 under hypoxia [36]. NF-kBp50 levels in P-PIC placentas increased 2.7 fold compared to P placentas (p<0.05, Fig. 1D **)** suggesting that NF-kBp50 also regulates miR-210 expression.

### STAT6 is a Target of miR-210

**Figure 2:**
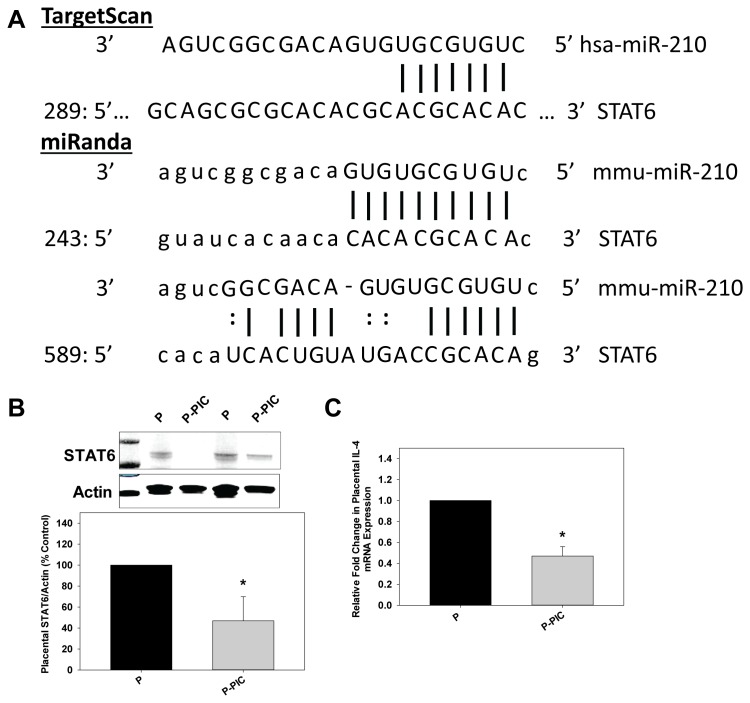
Placental STAT6 and IL-4 levels are decreased significantly in P-PIC mice. A. Schematic of human *STAT6* 3′-UTR sequence targeted by miR-210. The predicted seed regions by both TargetScan and miRanda algorithms are indicated. B. Placental cell lysates from both P and P-PIC mice at gestational day 18 were subjected to immunoblot analyses using anti-STAT6 antibodies. Immunoblot analysis with an anti-β-actin antibody served as a loading control. The first lane in the immunoblot indicates the molecular weight marker. C. mRNA levels of IL-4 were determined by qRT–PCR after isolation of total RNA from placentas of both P and P-PIC mice at gestational day 18. qRT–PCR of GAPDH was used for normalization. Results are expressed as mean+SEM for 3 independent experiments. *P<0.05 vs. P.

TargetScan [22] and miRanda [40] algorithms suggested STAT6 as a target of miR-210 (Fig. 2A). Targetscan predicts one site (P_ct_ < 0.1) whereas miRanda suggests two putative binding sites for miR-210 in the 3′UTR of STAT6 and the miRSVR score for binding site 1 is −0.587 and for binding site 2 is −0.0257. Since the 3′UTR of STAT6 is a predicted target of miR-210, we measured protein levels of STAT6. STAT6 levels were decreased significantly in the placentas of P-PIC mice compared to P mice (Fig. 2B). IL-4 binding to its own receptor activates STAT6 which in turn activates T helper (Th)-2 lineage-specific transcription factor GATA3. GATA3 then binds to the promoter of Th2 cytokines such as IL-4 to regulate their expression. Because STAT6 levels were decreased in P-PIC placentas, we sought to determine if IL-4 levels were negatively affected. IL-4 mRNA levels decreased significantly in placentas from P-PIC mice compared to P mice (Fig. 2C).

### miR-210 Expression in TLR3 Deficient Pregnant Mice Treated with Poly I:C

**Figure 3:**
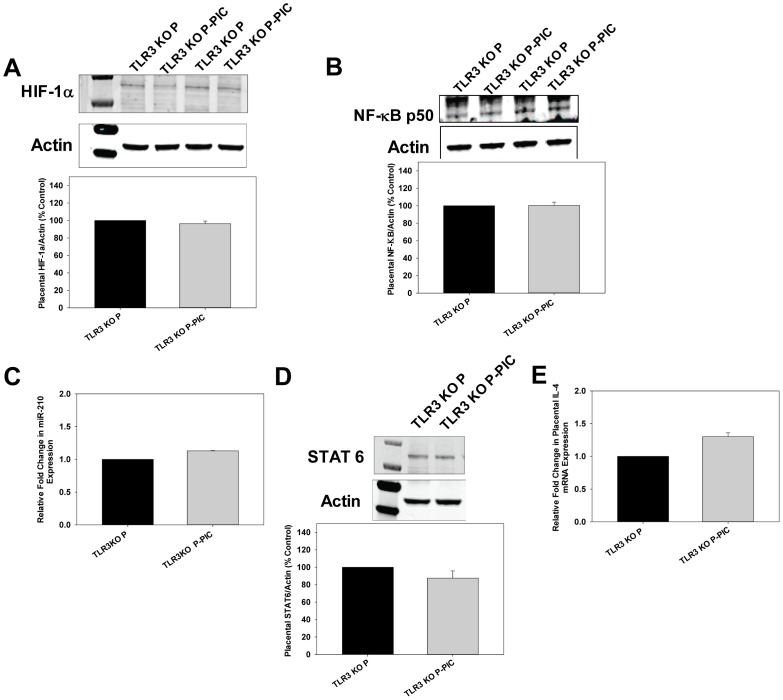
No significant change in placental HIF-1α, NF-κB, miR-210, STAT6, and IL-4 levels in TLR3 KO P-PIC mice. A and B. Placental cell lysates from both P TLR3 KO and P-PIC TLR3 KO mice at gestational day 18 were subjected to immunoblot analyses using anti-HIF-1α and anti-NF-κB antibodies. No significant difference was noted in HIF-1α and NF-κB levels between P TLR3 KO and P-PIC TLR3 KO mice. The first lane in the immunoblot A indicates the molecular weight marker. C. Placental miR-210 levels in both P TLR3 KO and P-PIC TLR3 KO mice were determined by qRT-PCR. D. No difference was noted in placental STAT6 levels between P TLR3 KO and P-PIC TLR3 KO mice by immunoblot analysis. The first lane indicates the molecular weight marker. Because we did not observe any significant difference in STAT6 levels between WT P and TLR3 KO P mice indicating that deficiency of TLR3 in mice did not alter baseline STAT6 levels we did not include this data in Fig. 3. E. IL-4 mRNA levels were determined by qRT–PCR from placentas of both P TLR3 KO and P-PIC TLR3 KO at gestational day 18. GAPDH was used for normalization. Results are expressed as mean+SEM for 3 independent experiments. *P<0.05 vs. P TLR3 KO.

In order to determine if TLR3 activation directly contributes to miR-210 up-regulation, we treated pregnant TLR3 KO mice with poly I:C (P-PIC TLR3 KO). Interestingly, these mice did not develop PE-like symptoms including hypertension, endothelial dysfunction, and proteinuria (data not shown). Placental HIF-1α and NF-κBp50 levels did not change between P-PIC TLR3 KO and P TLR3 KO mice (Fig. 3A and 3B). Consistent with the finding that neither of the transcriptional activators was increased, we did not observe any increase in placental miR-210 expression in P-PIC TLR3 KO mice (Fig. 3C). Similarly, placental STAT6 and IL-4 levels did not change in P-PIC TLR3 KO mice (Fig. 3D and 3E).

### TLR3 Induced miR-210 Up-regulation in Human CTBs

**Figure 4:**
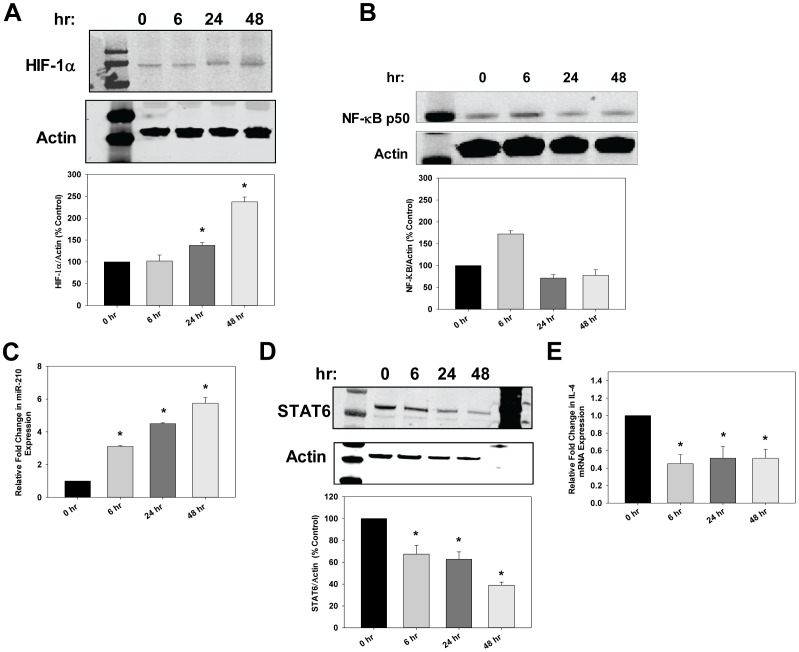
Poly I:C increased HIF-1α and NF-κB which induced miR-210 leading to decreased STAT6 and IL-4 in human CTBs. A and B. CTB cells were treated with poly I:C (2 ug/ml) for 6, 24, or 48 hrs. Cell lysates were isolated and immunoblotting was performed using anti-HIF-1α and anti-NF-κB antibodies. Results are expressed as mean+SEM for 3 independent experiments. The first lane in the immunoblots in A and B indicate the molecular weight marker. C. Under the same conditions as above, miR-210 expression increased following TLR3 activation with the peak induction seen at 48 hrs as determined by qRT-PCR. D. Poly I:C treatment for 48 hrs showed the strongest down-regulation of STAT6 as determined by immunoblotting. The first and last lanes in the immunoblot indicate the molecular weight marker. E. Relative fold-change in IL-4 levels following poly I:C treatment in CTB cells. Results are expressed as mean+SEM and *P<0.05 vs. P.

To determine the placental etiology we next treated human CTBs with poly I:C, which is effective up to 48 hrs [41], [42]. HIF-1α levels increased after 24 and 48 hrs of poly I:C treatment (Fig. 4A). NF-kBp50 levels increased at 6 hrs and returned to basal levels at 24 hrs (Fig. 4B). miR-210 expression was increased significantly at 6, 24, and 48 hrs after poly I:C treatment (Fig. 4C). These results indicate that the transcription factors may bind to the promoter at different time points and their interplay regulates the expression of miR-210. STAT6 and IL-4 levels decreased after 6 hrs of poly I:C treatment (Fig. 4D and Fig. 4E).

### Validation of STAT6 as a Target of miR-210 in CTBs

**Figure 5:**
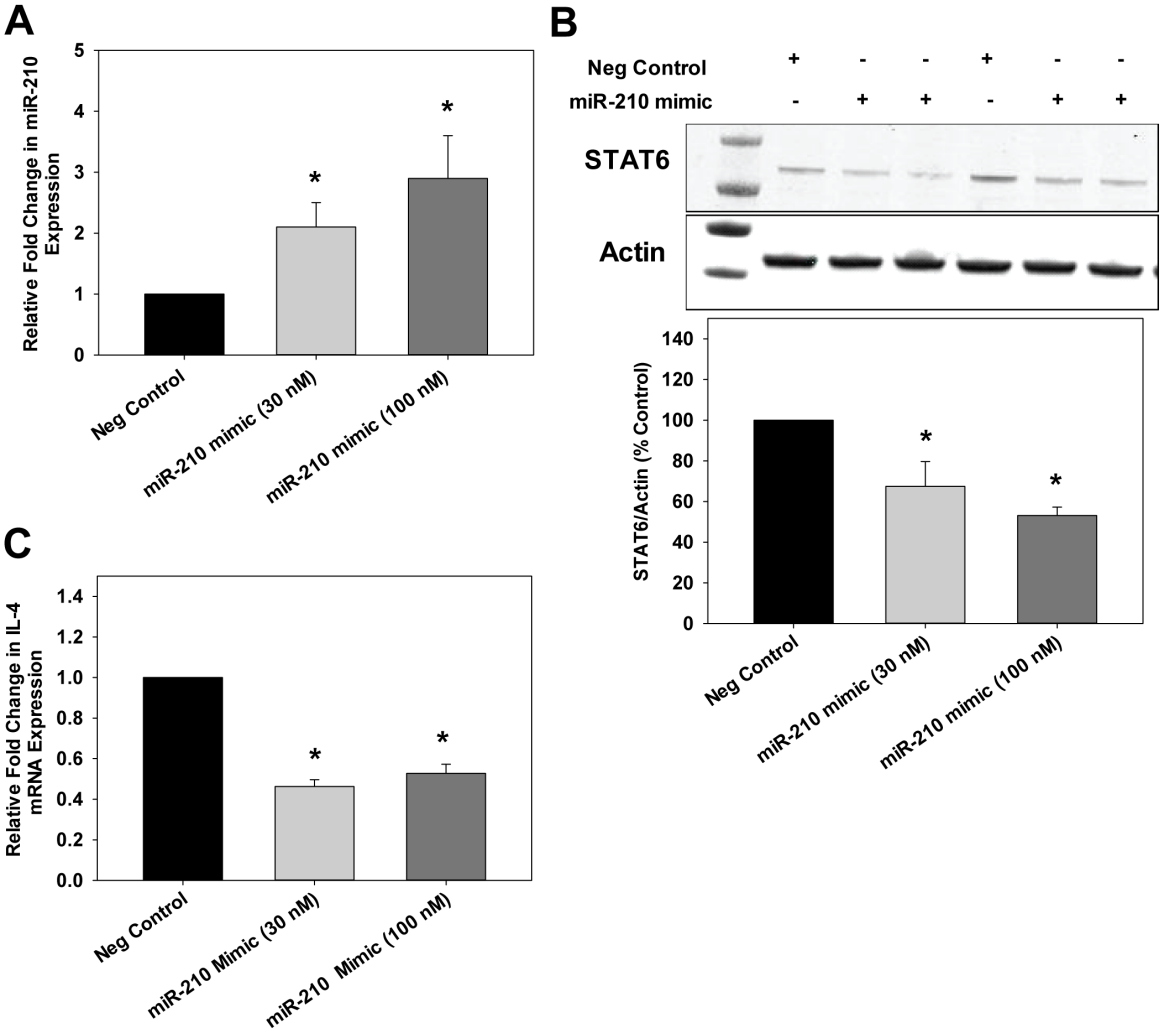
miR-210 mimic decreased STAT6 and IL-4 levels in human CTBs. CTBs were transfected with either scramble (negative control) or different concentrations of miR-210 mimic (30 nM or 100 nM). A. After 48 hrs of treatment, RNA was isolated and qRT-PCR was performed to determine miR-210 levels. U6 RNA was used as the normalization control. Results are expressed as mean+SEM for 3 independent experiments. B. Cell extracts were prepared and protein levels of STAT6 were determined by immunoblot. Overexpression of miR-210 decreased STAT6 levels compared to scramble (negative control). The first lane in the immunoblot indicates the molecular weight marker. C. Total RNA was isolated and IL-4 levels were determined by qRT-PCR. Relative expression of IL-4 was determined. Results are expressed as mean+SEM and *P<0.05 vs. scramble.

**Figure 6:**
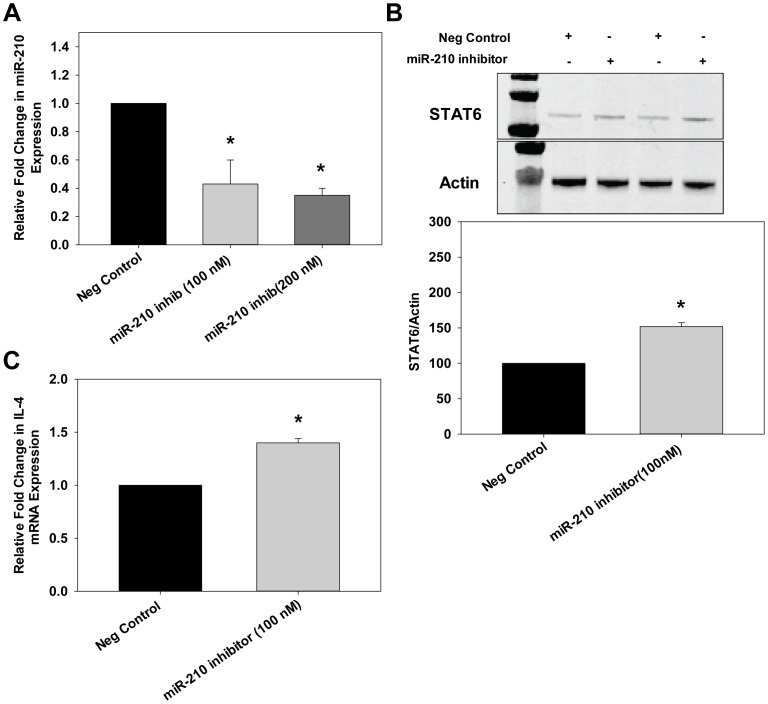
Anti-miR-210 increased STAT6 and IL-4 levels in human CTBs. CTB cells were transfected with either scramble (negative control) or different concentrations of a miR-210 inhibitor (100 nM or 200 nM). A. After 48 hrs of treatment, RNA was isolated and qRT-PCR was performed to determine miR-210 levels. U6 RNA was used as the normalization control. Results are expressed as mean+SEM for 3 independent experiments. B. Cell extracts were prepared and protein levels of STAT6 were determined by immunoblotting. Down-regulation of miR-210 increased STAT6 levels compared to scramble (negative control). The first lane in the immunoblot indicates the molecular weight marker. C. Total RNA was isolated and IL-4 levels were determined by qRT-PCR. Relative expression of IL-4 was determined. Results are expressed as mean+SEM and *P<0.05 vs. scramble.

To investigate the activity of miR-210, we transfected CTBs with Pre-miR™ miRNA precursors that are stable, chemically modified double-stranded RNAs that mimic mature endogenous miRs. We confirmed increased expression of miR-210 in CTBs transfected with a pre-miR mimetic of miR-210 by qRT-PCR (Fig. 5A). The pre-miR mimetic of miR-210 significantly reduced STAT6 levels by ∼40–50% as determined by immunoblot compared to the control, a random precursor (Fig. 5B). IL-4 expression also significantly decreased ∼40–50% compared to control (Fig. 5C). To further investigate the role of miR-210 on STAT6/IL-4 expression, anti-miR-210 was transfected into human CTBs. Inhibition of miR-210 was similar at either 100 or 200 nM thus we used 100 nM for all subsequent studies (Fig. 6A). After 48 hrs of transfection, a significant increase in STAT6 (Fig. 6B) and IL-4 (Fig. 6C) expression was observed after miR-210 inhibition. These results indicate a direct role of endogenous miR-210 in the negative regulation of STAT6 and IL-4.

## Discussion

PE is a multifactorial disease and the role miRs play in the pathophysiology of PE is only beginning to emerge. Here we demonstrate that TLR3 activation induces miR-210 expression via the transcription factors HIF-1α and NF-kBp50 and that STAT6 is a target of miR-210. The induction of miR-210 by TLR3 decreases STAT6 and IL-4 which may contribute to the inflammatory state evident in PE.

Pineles *et al.* published a study on the differential expression of miRs where 157 miRs were screened in both PE and normal placentas [29]. miR-210 was increased significantly in PE placentas and mostly localized in the syncytiotrophoblasts. Target genes of miR-210 were predicted to regulate immune responses, apoptosis, and lipid metabolism. Zhu *et al.* performed placental miR microarray analyses from both mild and severe PE patients. Thirty-four miRs were expressed differentially in PE placentas compared to normal placentas [31]. Of these, 11 miRs were overexpressed and miR-210 expression was increased 3.6-fold in severe PE patients. A study by Enquobahrie *et al.* indicated a significant 1.5-fold increase in miR-210 expression in PE placentas [43]. Our results demonstrate for the first time that TLR3 activation in human CTBs significantly increases miR-210 expression and in our TLR3-induced mouse model of PE placental miR-210 expression is increased significantly. Furthermore, P-PIC TLR3 KO mice, which exhibited no symptoms of PE, did not exhibit an increase in placental miR-210 expression compared to P TLR3 KO mice. Recent studies indicate that miR-210 is also up-regulated in the plasma of PE women [44]. Thus, increased miR-210 expression may serve as a potential biomarker for PE. Currently, only protein markers in plasma or serum are used for predicting PE [45], however one limitation is that none of the current detection methods are reliable predictors of PE. Levels of miR-210 may be a more accurate predictor of PE as miR-210 expression in serum samples increases with the severity of PE [44], but this remains to be determined.

The promoter of miR-210 has several putative transcription factor binding sites [34]. It has been previously shown that HIF-1α regulates the expression of miR-210 in a variety of tumor types through a hypoxia-responsive element [34]. In support, several studies showed that the expression of miR-210 increases upon exposure to hypoxia, which implies that miR-210 may be a potential marker of hypoxia. PE pregnancies are associated with poor placentation that can cause focal regions of ischemia/hypoxia in the placenta which in turn may cause increased miR-210 expression. In addition to hypoxia, HIF-1α expression is also induced under normoxic conditions as HIF-1α levels were up-regulated in the prostate cancer cell line PC3 but not LNCaP [46]. Our results demonstrate that HIF-1α levels were also increased in the placentas of P-PIC mice as well as normoxic CTBs treated with poly I:C in a time-dependent manner. HIF-1α induced under normoxic conditions may in turn bind to the miR-210 promoter and up-regulate its expression. Apart from the CTBs, whether or not HIF-1α up-regulation occurs in other cell types in the placenta following TLR3 activation remains to be determined. Recently, another transcription factor, the NF-κBp50 subunit, was also shown to bind the miR-210 promoter and regulate its expression [36]. We demonstrate that CTBs treated with poly I:C as well as placentas from P-PIC mice have elevated levels of NF-κBp50 whereas P-PIC TLR3 KO placentas exhibit no change in expression of NF-κBp50 or HIF-1α. NF-κB levels were also increased in placentas of PE women compared to normotensive women as detected by immunohistochemistry [47]. Taken together our data suggests that both of these transcription factors are up-regulated following TLR3 activation and that their interplay contributes to miR-210 transcriptional up-regulation.

Several targets of miR-210 have been identified through a combination of target prediction algorithms and biochemical assays. So far, hydroxysteroid (17-β) dehydrogenase 1, ISCU, Ephrin A3, and HOXA9 have been identified in PE [36], [38], [39].

The role of miR-210 in angiogenesis and iron metabolism are well established but the role of miR-210 in the regulation of genes related to immune responses are unknown. Pineles *et al.* used miRBase to determine putative mRNA targets of miR-210 and miR-210 targets are enriched in genes related to immune responses [29]. Of the several immune related targets, we demonstrate using overexpression and inhibition studies that STAT6 is a bona-fide target of miR-210.

IL-4 binds IL-4 Receptor α (IL-4Rα) and activates STAT6 leading to increased expression of the GATA3. GATA3 binds to target regulatory sequences of Th2 cytokine genes including IL-4 thus promoting expression [48]. We have shown that IL-4 levels fail to increase in the serum of P-PIC mice which exhibit inflammation and PE-like symptoms [13]. These results taken together suggest that IL-4 plays an important role in maintaining immune system homeostasis during pregnancy. In this paper we demonstrate that IL-4 expression in placentas of P-PIC mice and in CTBs decreased significantly following TLR3 activation. Moreover, overexpression of miR-210 in CTBs decreased IL-4 expression and vice-versa suggesting that decreased levels of STAT6 contribute to the decrease in IL-4 expression.

In summary, our work suggests that miR-210 expression is increased by TLR3 possibly via HIF-1α and NF-κB leading to decreased levels of STAT6 and IL-4 and may partly contribute to the development of PE. Several recent papers demonstrate the efficacy of administration of antimiR oligos. Among them, a miR-208a antimiR has been shown to suppress fibrosis by reducing myosin 7 expression and improve survival in Dahl salt-sensitive rats [49]. miR-21 inhibitors were effective in suppressing extracellular matrix production in several diseases such as muscular dystrophy, renal fibrosis, and pulmonary fibrosis [50]–[52]. Furthermore, miR-122 antimiR oligos have been successfully used for the treatment of HCV in recent Phase I and Phase II clinical trials. Therefore, miRs inhibitors may be a promising therapeutic for a variety of diseases. Based on our results, miR-210 inhibition may ameliorate the symptoms of PE.

## Materials and Methods

### Animals/Treatments

Male breeder mice, female mice (C57BL/6J) stock #002253, and TLR3^+/−^ mice (B6;129S1-*Tlr3^tm1Flv^*/J) stock #005217 were obtained from The Jackson Laboratory. TLR3^+/−^ mice were intercrossed to generate TLR3^−/−^ mice and used for the studies reported here. All mice were acclimated in our facility and housed in a temperature-controlled room (23°C) with a 12-h:12-h light-dark cycle. Adult male and female mice (8–12 weeks old) were mated and female WT or TLR3 KO mice were grouped either as pregnant (P) or pregnant treated with poly I:C (P-PIC**)**. Only P-PIC mice in both WT and TLR3 KO groups received i.p. injections of poly I:C (20 mg/kg) on gestational days 13, 15 and 17 as described previously [13], [14]. P mice in both WT and TLR3 KO groups received injections of saline (vehicle) on corresponding days. Both WT and TLR3 KO pregnant female mice treated with saline/poly I:C were euthanized on gestational day 18 and organs were harvested, snap frozen, and stored at −80°C for subsequent studies. All animals were maintained in the Scott & White Healthcare Animal Facility under specific pathogen-free conditions. All experimental procedures were approved by the Texas A&M Health Science Center/Scott & White Healthcare Institutional Animal Care and Use Committee in accordance with the NIH Guide for the Care and Use of Laboratory Animals.

### Blood Pressure Measurements

Systolic arterial blood pressure was measured in conscious animals using non-invasive, computerized tail-cuff plethysmography (IITC, Inc.) as described previously [53]. Mice were acclimatized for 3 days by placing them in the restrainer prior to data collection. Measurements were taken at baseline and on gestational days 13 and 17 prior to injections on that day. The average of 3 measurements was used for data analysis.

### Cell Culture Studies

The human extravillous CTB cell line, SGHPL-4, was derived from first trimester chronic villous tissue and provided by Dr. Guy Whitley (St. George’s Hospital Medical School, London, UK) [54]. SGHPL-4 cells were cultured in Ham’s F-10 media supplemented with 10% fetal bovine serum, penicillin G, streptomycin, and l-glutamine (Sigma). Cells were grown at 37°C in 5% CO_2_.

### Immunoblots

Cell lysates were prepared from mouse placental tissues and human CTBs using a cell lysis buffer (Cell Signaling) with protease inhibitors and subjected to electrophoresis with SDS–PAGE (4–12%) gels. The blots were transferred onto nitrocellulose membranes (Millipore) and Western blot analyses were performed using the following primary antibodies: STAT6 1:1,000 (Cell Signaling), HIF-1α 1∶500 (Novus Biologicals for mouse), HIF-1α (R&D Systems for human), and NF-κB 1∶1000 (Cell Signaling). Beta-actin 1∶10,000 (Sigma) was used as a loading control. The secondary antibodies consisted of anti-rabbit and anti-mouse IgGs conjugated to Alexa-Fluor 680 or IRDye 800 (LI-COR Biosciences). Infrared visualization was used followed by densitometric analyses using the provided software (Odyssey System, LI-COR Biosciences).

### qRT-PCR

Total RNA (including miRs) from placentas and CTBs was extracted using an miRNeasy isolation kit (Qiagen) and quality and quantity was determined using a NanoDrop spectrophotometer. miRs were further isolated from total RNA using an RT^2^ qPCR-grade miRNA isolation kit (SABiosciences). cDNA was prepared using a RT^2^ miRNA First Strand kit (SABiosciences) and miRNA expression was assessed by real-time PCR using SYBR Green (SABiosciences). The expression of miR-210 was normalized to mouse snoRNA 142 for mouse placentas and to human U6 for CTBs. All the above-mentioned primers were obtained from SABiosciences. To determine IL-4 mRNA levels, first-strand cDNAs were synthesized with reverse transcriptase (Qiagen) using total RNA (1 µg) extracted with an RNeasy kit (Qiagen) and amplified using a SYBR Green PCR master mix (SABiosciences) per the manufacturer’s protocol. Primers used for human IL-4 and GAPDH were purchased from SABiosciences. The forward and reverse primers for the mouse IL-4 gene that were used are- Forward primer: 5′ GGT CAC AGG AGA AGG GAC GCC 3′ Reverse primer: 5′ TGC GAA GCA CCT TGG AAG CCC 3′ and the GAPDH mouse primers are- Forward primer- 5′ TCA CCA CCA TGG AGA AGG C 3′ Reverse primer- 5′ GCT AAG CAG TTG GTG GTG CA 3′. Reactions were carried out in a 96-well optical reaction plate using a Real-Time PCR Detection System (Stratagene, Mx3000p). Reactions proceeded with an initial 10 min incubation at 95°C followed by 40 cycles of amplification: 95°C for 15 sec and 60°C for 1 min. Dissociation curves for each primer were verified to have single peaks. Comparative quantitation was performed by comparing the *C*_t_ value obtained and relative miRNA/mRNA abundance was calculated using the −ΔΔ*C*_t_ method [55].

### miR-210 Overexpression and Inhibition

Transfection of miR-210 mimics (pre-miRs; Dharmacon) or miR-210 inhibitors (anti-miRs; Ambion) was performed in human CTBs using the reverse transfection technique recommended for use with the siPORT *NeoFX* transfection reagent (Life Technologies). Control scramble sequence was used for each transfection reaction. Transfections were carried out when cells were 70% confluent according to the manufacturer’s protocol using 3 µL of transfection reagent and a final concentration of 30, 100, or 200 nM of each oligonucleotide per well in a 6-well plate. Media was replaced after 16 hrs and experiments were performed 24 hrs later.

### Statistical Analyses

Data are presented as mean ± SEM. SigmaStat 12 (Systat Software) was used to perform all statistical analyses. Student’s t-test was used for analyses within human CTBs and an ANOVA was used for comparisons between groups of mice for all measures followed by the Student-Newman-Keuls *post hoc* test. The significance level was 0.05.
